# Coliform Load and Antimicrobial Resistance in Ghana’s Seafood Processing Effluent (2021–2024): Evidence of Operational Improvement and Persistent AMR Risk

**DOI:** 10.3390/life16010107

**Published:** 2026-01-12

**Authors:** Ebenezer Worlanyo Wallace-Dickson, Meldon Ansah-koi Agyarkwa, Nana Ama Browne Klutse, Esi Nana Nerquaye-Tetteh, Abdalla Abubakari, Selina Amoah, Jewel Kudjawu, Godfred Saviour Azaglo, Mariam Fuowie Batong, Johnson Ade, Isaac Junior Okyere, Mary-Magdalene Osei, Karyn Ewurama Quansah, Emmanuel Martin Obeng Bekoe, George Kwesi Hedidor, Divya Nair, Robert Fraser Terry, Japheth A. Opintan

**Affiliations:** 1 Environmental Protection Authority, Starlets ’91 Road, Ministries, Accra P.O. Box MB 326, Ghana; meldon.agyarkwa@epa.gov.gh (M.A.-k.A.); nana.klutse@epa.gov.gh (N.A.B.K.); esi.nerquaye-tetteh@epa.gov.gh (E.N.N.-T.); abdalla.abubakari@epa.gov.gh (A.A.); selina.amoah@epa.gov.gh (S.A.); jewel.kudjawu@epa.gov.gh (J.K.); saviour.azaglo@epa.gov.gh (G.S.A.); mariam.batong@epa.gov.gh (M.F.B.); johnson.ade@epa.gov.gh (J.A.); 2 Department of Chemistry, SUNY College of Environmental Science and Forestry, Syracuse, NY 13210, USA; iokyere@esf.edu; 3 Department of Medical Microbiology, University of Ghana Medical School, Accra P.O. Box GP 4236, Ghana; mmosei@ug.edu.gh (M.-M.O.); jaopintan@ug.edu.gh (J.A.O.); 4 Council for Scientific and Industrial Research-Water Research Institute (CSIR-WRI), Achimota, Accra P.O. Box AH 38, Ghana; karynquansah@gmail.com (K.E.Q.); emobekoe@csir.org.gh (E.M.O.B.); 5 World Health Organization Country Office, Roman Ridge, Accra P.O. Box MB 142, Ghana; hedidorg@who.int; 6 Independent Researcher, Scottsdale, AZ 85259, USA; divsnair08@gmail.com; 7 World Health Organization Special Programme for Research and Training in Tropical Diseases (TDR), World Health Organization, 1211 Geneva, Switzerland; terryr@who.int

**Keywords:** SORT IT, One Health, operational research, antimicrobial resistance, effluent, seafood processing, potable water

## Abstract

Antimicrobial resistance (AMR) can disseminate through effluents from seafood processing facilities (SPFs), posing environmental and public health risks. This study assessed changes in coliform load and antimicrobial resistance patterns in effluents from two SPFs in Tema, Ghana, before and after upgrades to effluent treatment systems between 2022 and 2024. A total of 19 effluent samples were collected per SPF in 2021–2022, 20 effluent samples each per SPF in 2024, and 8 potable water samples each per SPF in 2024. Median coliform counts declined significantly in both facilities (SPF-1: 920 to 35 MPN/100 mL; SPF-2: 280 to 9.5 MPN/100 mL; *p* < 0.001), representing a 96% overall reduction. *Escherichia coli* prevalence decreased markedly in SPF-2, although *Pseudomonas aeruginosa* emerged after treatment upgrades. Resistance to third-generation cephalosporins and multidrug resistance declined, particularly in SPF-1, but persisted across both facilities. Potable water used for seafood processing showed low but detectable coliform contamination. Despite substantial reductions in coliform bacterial load, the continued presence of resistant gram-negative bacteria highlights the need for sustained AMR surveillance, mandatory effective effluent treatment, and routine disinfection of potable water to protect public health.

## 1. Introduction

Antimicrobial resistance (AMR) is a global health challenge, as infections caused by drug-resistant microorganisms can lead to increased deaths, facilitate transmission to others, and create substantial economic and social burdens. The situation is particularly critical in low and middle-income countries (LMICs), where constrained healthcare systems and limited resources intensify its impact [1]. To combat AMR, the World Health Organization (WHO) has emphasized the “One Health” approach [2]. This approach includes humans, animals, the environment, and the interconnections between them as one entity. Surveillance for certain priority pathogens that pose significant threats across human, animal, and environmental health systems is an essential component of combating AMR from a One Health approach [3].

Drug-resistant microbes can reach the human environment interface through different routes. Seafood processing facilities (SPFs) are environments where antibiotic-resistant bacteria can develop and spread [4,5]. It is recognized that the initial washing, butchering, and general cleaning of the processing equipment in the SPFs consume a significant amount of water [6]. The effluents released after these processes are rich in organic by-products of seafood processing and can be a nutrient-rich source for microbial development [7]. The bacterial load in effluents discharged from SPFs can also be increased by using ice and water tainted with microorganisms during post-harvest processing and storage. [8]. Numerous gram-negative bacterial taxa have been isolated from surfaces that come into contact with seafood, including *Enterobacter*, *Klebsiella*, *Pseudomonas*, *Aeromonas*, *Alcaligenes*, and *Vibrio.* [9,10,11]. Studies from different parts of the globe have reported multidrug-resistant *Escherichia coli* (*E. coli*), *Proteus* spp., and *Vibrio* spp. in effluents, which indicates high contamination and improper treatment of effluents discharged from the SPFs [12,13,14,15].

In Ghana, the fisheries sector is a cornerstone of the national economy, contributing to livelihoods, food security, and export earnings, with numerous seafood processing facilities (SPFs) operating along the coastline [16].

Recognizing the growing threat of AMR, Ghana instituted a five-year National Action Plan on AMR in 2017, which emphasized the need for enhanced AMR surveillance across sectors, including the aquaculture and fisheries industry [17]. The plan aligns with the WHO’s Global Action Plan (GAP) on AMR and advocates a One Health approach, reinforcing collaboration among sectors such as the Ministry of Fisheries and Aquaculture Development, the Environmental Protection Authority (EPA), and the Food and Drugs Authority [18].

An operational research (OR) study conducted in 2021–2022 reported high levels of coliform bacterial load and AMR bacteria in effluents from two SPFs in Tema, Ghana [19]. Additionally, the investigation found that certain effluent samples had WHO-priority microorganisms, most notably *E. coli* resistant to cefotaxime, ceftazidime, and meropenem, the most potent antibiotics currently available for the treatment of infections caused by these bacteria. Subsequently, EPA met with the facility managers of the SPFs to review their effluent treatment systems and adopt best practices to prevent the emergence and spread of AMR. The baseline OR study did not ascertain whether the potable water used for processing the seafood could also be a source of AMR.

In this context, a follow-up observational study was conducted in 2024 to compare the operational characteristics of two seafood processing facilities between 2021–2022 and 2024, including the types of seafood processed and the volumes and sources of water used during production, as reported by facility managers. This study further evaluated changes in coliform bacterial loads and the burden of WHO-priority gram-negative pathogens in effluents discharged from the same facilities in Tema, Ghana, relative to the baseline study. In addition, coliform bacterial loads in potable water used for seafood processing were assessed. The findings of this comparative study can inform the development of guidelines and standards for discharge of effluents and modalities for AMR surveillance in the environment sector including fisheries.

## 2. Materials and Methods

### 2.1. Study Design

This study employed a repeated cross-sectional pre–post design to compare coliform load and antimicrobial resistance indicators in two seafood processing facilities between 2021—2022 and 2024. Ghana is a nation in the West African sub-region that is situated between the Atlantic Ocean and the Gulf of Guinea. There are roughly 30.8 million people living in the nation, with Greater Accra being the most populous area [20].

### 2.2. Study Settings

Ghana’s Atlantic Coast is home to the city of Tema. It is situated 25 km east of Accra, the nation’s capital. Tema is home to Ghana’s biggest seaport. Tema has SPFs that make canned seafood, primarily tuna. Potable water used in seafood processing at both facilities was supplied by Ghana Water Limited (GWL), Tema Region, Ghana. Where applicable, the supplied water underwent routine on-site handling (e.g., storage and distribution within the facility) prior to use in production processes. Wastewater generated during processing was collected and treated through the facility effluent treatment systems before final discharge into the receiving open drain leading to the Chemu Lagoon. The amount of water consumed in SPF-1 was 669,000 L in 2021 and 645,000 L in 2024. In SPF-2, the water consumption was 1,220,180 L in 2021 and 1,047,500 L in 2024. These SPFs employ an anaerobic and aerobic effluent treatment system that uses polymeric coagulants to lower the bacterial burden. Open community sewers and the adjacent Chemu Lagoon, which empties into the Atlantic Ocean, receive the effluent discharge from SPF-producing operations. Sometimes irrigation is conducted using water from the Chemu Lagoon.

This study focused on two large SPFs (referred to as SPF-1 and SPF-2 in the rest of the paper) in Tema, which have the EPA’s permit to operate. Both of the SPFs mainly process tuna. The average daily fish production capacity was 140 metric tons for SPF-1 and 130 metric tons for SPF-2 in 2024. SPF-1 was functioning at reduced production capacity in 2024 due to ongoing renovation and overhaul of their effluent treatment systems.

In Ghana, the EPA recognizes the need to reduce pollution to levels that minimize harmful effects on human health and the environment. In view of this, the EPA monitors industries with the potential to be hotspots for growth of AMR bacteria, including pharmaceutical industries, fish processing, slaughterhouses, etc. As part of the routine AMR surveillance by the EPA, effluent samples are collected on a quarterly basis from SPFs. These samples are analyzed at a central laboratory for coliform bacterial load, bacterial species, and their AMR profile. These data are used to generate quarterly and annual environmental quality reports by the EPA. These reports form the basis for making recommendations to improve the operations of the SPFs and ensure minimal adverse impact on the environment.

### 2.3. Baseline Study, Dissemination Activities, Recommendations, and Actions Taken

In October 2022, a special Structured Operational Research and Training Initiative (SORT IT) workshop was held to build the practical abilities and resources needed to effectively convey the baseline OR study’s findings. These tools included the following: (1) A communication plan to target decision-makers and stakeholders; (2) a one-page plain language summary of the key messages written in a short and simple manner; (3) PowerPoint presentations to be used at national fora and conferences; and (4) an elevator pitch—a one-minute oral presentation for opportunistic one-to-one conversations.

Supplementary Table S1 provides details about, how, to whom, where, and when, the findings of the baseline operational research study conducted by Agyarkwa et al., 2022 [19], were disseminated.

### 2.4. Recommendations, Action Status, and Details of Action

Following the baseline OR research study in 2021/2022, some recommendations, action, and specific activities for improving seafood processing methods were recommended to SPF-1 and SPF-2 by the EPA.

EPA-Ghana engaged the management of the two SPFs to review and rectify their treatment system to ensure reduction in coliform load in their effluent. This action was implemented by two SPFs where they considered revamping the whole effluent treatment system to include clarifiers and dissolved air flotation (DAF) system as highly effective in removing fine suspended solids, fats, oils, and grease which are not easily removed by gravity and sedimentation thus reducing the organic matter content in the effluent. System in addition to the use of ferric chloride. SPF-1 introduced polyacrylamide (PAM) as a more effective coagulant for treating their effluents before discharge into the environment in 2023.

### 2.5. Study Population and Study Period

In the baseline OR study, 19 samples of effluent were collected from each SPF between May–July 2021 and April–May 2022 on a weekly basis. In the follow-up OR study, 20 effluent samples were collected from each SPF within a two-week interval between March to November 2024. Additionally, in the follow-up study, 8 samples of potable water, that is, water used in their production processes, were also collected from each SPF between March and November 2024. Samples for the baseline OR study were collected and analyzed during May–July 2021 and March–May 2022. The samples for the follow-up study were collected and analyzed during March–November 2024. Data analysis and compilation of results were conducted in April 2025.

### 2.6. Sample Collection and Laboratory Analysis

Potable water used for seafood processing was collected from taps located in the production rooms of each seafood processing facility (SPF). Effluent samples were collected from the final discharge points of the facilities, which discharge into an open drain flowing into the Chemu Lagoon, following the same sampling approach described by Agyarkwa et al. (2022) [19]. Sampling was conducted according to the same schedule and at comparable times at both facilities to ensure consistency with the baseline study. The Most Probable Number (MPN) technique was used for sample collection and analysis in compliance with the guidelines provided in the Standard Methods for the Examination of Water and Wastewater (2012) [21]. Ambient water temperature and pH were measured in situ at the time of collection. Effluent and potable water grab samples were aseptically collected into sterile, labelled 1-liter high-density polyethylene (HDPE) bottles and transported on ice packs to the Department of Medical Microbiology Laboratory, University of Ghana Medical School, for analysis. Sampling was conducted at two-week intervals from March to November 2024, and all samples were analyzed within two hours of collection.

Before sample collection, the bottles were rinsed with an adequate amount of the effluent to condition them. The samples were preserved with ice packs in an ice chest before and during transport [22].

Lauryl Tryptose Broth (LTB) was used as the presumptive medium in the usual multiple-tube fermentation procedure used to test the samples [21]. For each positive sample, a single presumptive colony per target gram-negative bacterial species was selected based on colony morphology on selective media and sub-cultured for confirmation and antimicrobial susceptibility testing. Multiple colonies of the same species from a single sample were not included in order to reduce redundancy and minimize non-independence among isolates. Antimicrobial resistance analyses were therefore conducted at the isolate level, with each isolate corresponding to a distinct sample–species combination. Identification was accomplished using the BD Bruker MALDI Biotyper CA system (MBT compass for Research and MBT compass IVD for clinical diagnosis-Franklin Lakes, NJ, USA/Bruker Daltonics, Bremen, Germany) Matrix-Assisted Laser Desorption Ionization Time-of-Flight (MALDI-TOF) mass spectrometry [23]. Representative MALDI-TOF spectra is provided in Supplementary File S1.

The following gram-negative pathogens were the isolates of interest in this study: (a) *Enterobacteriaceae* (*E. coli*, *Klebsiella pneumoniae (K. pneumoniae)*, *Proteus* spp., *Shigella* spp. and *Salmonella* spp.); (b) *Pseudomonas aeruginosa (P. aeruginosa)*; (c) *Acinetobacter baumannii (A. baumannii);* (d) *Vibrio* spp. The Kirby–Bauer disk diffusion technique was used to test for antibiotic susceptibility in accordance with Clinical Laboratory Standards Institute (CLSI) standards [24,25]. A representative agar plate illustrating the assay is provided in the Supplementary Materials (Figure S1).

Sixteen antibiotics that are frequently used in Ghana were examined. Tetracycline (30 µg), sulfamethoxazole/trimethoprim (25 µg), gentamicin (10 µg), amikacin (30 µg), ampicillin (10 µg), amoxicillin/clavulanic acid (20/10 µg), ciprofloxacin (5 µg), levofloxacin (5 µg), ceftazidime (30 µg), piperacillin/tazobactam (10 µg), cefepime (30 µg), ceftriaxone (30 µg), meropenem (10 µg), and colistin (10 µg) were among them. Isolates that screened positive for Extended-Spectrum β-Lactamase (ESBL) or carbapenemase activity were subjected to confirmatory phenotypic assays. ESBL production was confirmed using the Combination Disk Test (CDT), in which the inhibition zones of cephalosporin disks (ceftazidime and cefotaxime) were compared with those of the corresponding disks containing clavulanic acid. An increase of ≥5 mm in zone diameter in the presence of clavulanic acid was interpreted as indicative of ESBL production. A representative ESBL-positive result is shown in the Supplementary Materials (Figure S2).

Carbapenemase activity was verified using the Modified Carbapenem Inactivation Method (mCIM), following standard CLSI (Clinical and Laboratory Standards Institute) protocols [24]. Carbapenemase production in *Escherichia coli* and *Klebsiella pneumoniae* isolates were confirmed using the mCIM according to CLSI guidelines [26]. Briefly, one to two colonies of each test isolate were emulsified in 2 mL of tryptic soy broth (TSB), after which a 10 µg meropenem disk was immersed in the suspension and incubated for 4 h at 35 ± 2 °C. Following incubation, the disk was removed and placed on a Mueller–Hinton agar plate inoculated with a 0.5 McFarland suspension of *E. coli* ATCC 25922 (carbapenem-susceptible indicator strain). Plates were incubated for 18–24 h at 35 ± 2 °C, and zones of inhibition were measured. Carbapenemase production was interpreted as positive when the zone of inhibition was 6–15 mm or when colonies were present within a 16–18 mm zone; negative when ≥19 mm; and indeterminate when 16–18 mm without colonies. To differentiate metallo-β-lactamases (MBLs) from serine carbapenemases, the ethylenediaminetetraacetic acid (EDTA)-modified CIM (eCIM) was performed in parallel. The procedure was identical to mCIM except that EDTA was added to the suspension prior to disk incubation. An increase in zone diameter of ≥5 mm with EDTA compared to mCIM indicated metallo-β-lactamase (MBL) production. The manufacturer’s instructions were followed in the preparation of every media. In accordance with 2021 CLSI recommendations, media and antibiotics were quality checked using the reference organism *E. coli* ATCC 25922 [27].

### 2.7. Data Collection, Source of Data, and Validation

A laboratory notebook was used to record the sample identifier, date of collection, coliform bacterial counts in MPN per 100 mL, bacterial species isolated for effluent samples, and the outcomes of the antibiotic susceptibility test. The Department of Medical Microbiology Laboratory at the University of Ghana Medical School then entered the data into an electronic database (Microsoft Excel). The EPA Environmental Quality Laboratory database maintains and shares this electronic database. The raw data from the laboratory notebook was used to validate the data in this study’s electronic database.

### 2.8. Data Analysis

Data required for this study were extracted from the laboratory database and entered on the Epicollect5 mobile application. Data from Epicollect5 were downloaded as an MS Excel Spreadsheet and imported into STATA version 16 (StataCorp, College Station, TX, USA) for analysis.

The coliform bacterial load in effluents and potable water samples collected at different time points was summarized in terms of the median and interquartile range (IQR) of the Most Probable Number (MPN). The median coliform bacterial load in the effluent samples between the two time periods were compared using the Mann–Whitney U test for both SPFs.

The number and proportion of samples in the two time periods that contained any of the gram-negative bacteria of interest and the WHO-priority pathogens (Carbapenem-resistant *P. aeruginosa*, fluoroquinolone-resistant *Salmonella* spp., fluoroquinolone-resistant *Shigella* spp.

Carbapenem-resistance *Enterobacteriaceae* carbapenem-resistant *A. baumannii*, *Enterobacteriaceae* resistant to 3rd generation cephalosporins, and *Vibrio* spp.) were summarized using percentages [28].

An isolate was classified as multidrug-resistant (MDR) if it showed phenotypic resistance to three or more different antimicrobial classes. The number and proportion of MDR isolates were reported out of the total number of isolates for both time periods for the two SPFs.

The proportion of samples with pathogens, resistant isolates, and MDR isolates were compared between the two time periods using the z-test for proportions or Fisher’s exact test (when expected numbers were small) for both SPFs. The level of significance was set at *p* ≤ 0.05. For those estimates where a statistically significant change was noted, the 95% confidence interval for the change was also calculated.

## 3. Results

A total of 19 effluent samples per SPF were collected for the baseline OR study between May–July 2021 and May–July 2022 whereas a total of 20 effluent samples per SPF were collected for the follow-up study between March and November 2024. Also, eight potable from each SPF used for seafood processing were also collected and analyzed for the follow-up study.

### 3.1. Median Coliform Bacterial Load in the Effluent Samples from Seafood Processing Facilities

**Figure 1:**
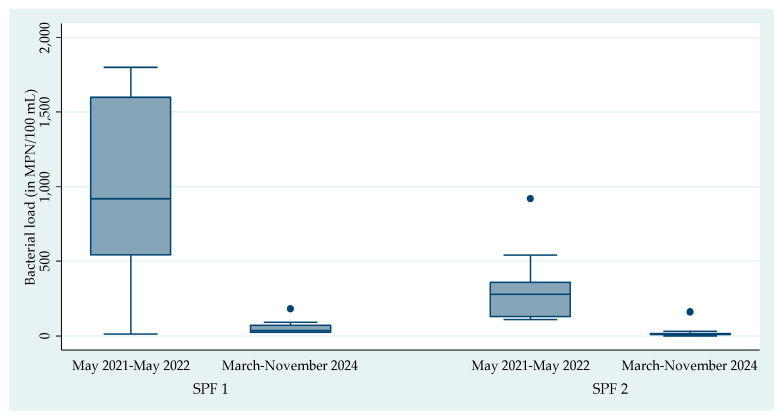
Median coliform bacterial loads in the effluent samples collected from the two seafood processing facilities in Tema, Ghana during May 2021–May 2022 in comparison to March–November 2024. Abbreviations: SPF- Seafood processing facility; MPN: Most probable number; *p*-value: SPF-1 (May 2021–May 2022 vs. March–November 2024) by Mann–Whitney U test is <0.001; *p*-value: SPF-2 (May 2021–May 2022 vs. March–November 2024) by Mann–Whitney U test is <0.001.

For SPF-1, the median coliform bacterial load significantly decreased from 920 MPN per 100 mL (IQR: 540–1600) in 2021–2022 to 35 MPN per 100 mL (IQR: 25–70) in 2024. Similarly, in SPF-2, the median coliform bacterial load dropped from 280 MPN per 100 mL (IQR: 130–360) to 9.5 MPN per 100 mL (IQR: 5–15) over the same periods (Figure 1). These reductions were statistically significant, with *p*-values < 0.001 in both facilities.

### 3.2. Detection of Gram-Negative Pathogens in Effluent Samples

In SPF-1, the proportion of effluent samples with *E. coli* increased from 84% (16/19 samples) in 2021–2022 to 95% (19/20 samples) in 2024. Samples with *Proteus* spp. isolates rose from 32% (6/19 samples) to 50% (10/20 samples). The increase in *E. coli* and *Proteus* spp. was not statistically significant. *Salmonella* spp. was not detected in 2021–2022 but was detected in three samples in 2024 (Table 1).

As shown in Table 1, SPF-2 showed a significant decline in *E. coli*, from 79% (15/19 samples) in 2021–2022 to 15% (3/20 samples) in 2024 (Decline: 64% (95% CI: 39% to 88%), *p* < 0.001). *P. aeruginosa* was absent in 2021–2022 but detected in 30% (6/20 samples) of 2024 samples (95% CI: 10% to 50%, *p* = 0.020).

None of the samples from either SPF during either of the two time periods included *Shigella* or *Vibrio* species.

### 3.3. Detection of WHO Priority Pathogens in Effluent Samples

In SPF-1, carbapenem-resistant *Enterobacteriaceae* were detected in one sample (5%) during each surveillance period. However, a significant reduction was observed in the detection of *Enterobacteriaceae* resistant to 3rd generation cephalosporins, from 37% (7/19 samples) in 2021–2022 to 5% (1/20 samples) in 2024 (Decline: 32% (95% CI: 8% to 55%), Fisher’s exact *p* = 0.020).

In SPF-2, carbapenem-resistant *Enterobacteriaceae* were not detected in 2021–2022 but appeared in one sample (5%) in 2024, though the difference was not statistically significant (*p* = 0.323). The proportion of samples with Enterobacteriaceae resistant to 3rd generation cephalosporins was 10% in both periods. Isolates that showed *Enterobacteriaceae* resistance to 3rd generation cephalosporins and carbapenems were subjected to antimicrobial susceptibility assays. Two (2) isolates of *K. pneumoniae* from SPF-2 and one (1) isolate of *E. coli* from SPF-1 were found to possess AmpC β-lactamase enzymes and ESBL. Carbapenem confirmation assays (mCIM) were negative, which implies isolates are resistant due to a non-carbapenemase mechanism.

The other WHO priority pathogens, namely carbapenem-resistant *P. aeruginosa*, fluoroquinolone-resistant *Salmonella* spp., fluoroquinolone-resistant *Shigella* spp., and carbapenem-resistant *A. baumannii*, were not detected in either of the SPFs.

### 3.4. Antibiotics Resistance Pattern in Bacterial Isolates from Effluent Samples

In both SPFs, the percentage of isolates of *E. coli* which showed multidrug resistance decreased by half (63% to 32% in SPF-1; 67% to 33% in SPF-2). There was also a reduction in the percentage of MDR *K. pneumoniae* isolates (40% to 25% in SPF-1, 100% to 56% in SPF-2) in both SPFs (Table 2). Pathogen occurrence is reported at the sample level, whereas antimicrobial resistance and multidrug resistance patterns are reported at the isolate level, with one isolate per species per sample.

Antibiotic resistance patterns varied across species and facilities. In SPF-1, *E. coli* resistance to tetracycline declined from 81% to 26% and to trimethoprim–sulfamethoxazole from 56% to 21%, while cefotaxime resistance dropped from 38% to 5%. In SPF-2, *E. coli* resistance to tetracycline (67% to 0%) and trimethoprim-sulfamethoxazole (53% to 0%) was eliminated, although ampicillin resistance remained high (87% to 67%). For *K. pneumoniae*, ampicillin resistance decreased from 80% to 0% in SPF-1, with cefuroxime resistance falling from 80% to 25%, but in SPF-2, resistance persisted, with new resistance to meropenem (11%) and ceftriaxone (11%) emerging in 2024. *Proteus* spp. in SPF-1 showed stable resistance to tetracycline (50%) and moderate resistance to ampicillin (40%). In SPF-2, the single *A. baumannii* isolate exhibited 100% resistance to multiple cephalosporins. Colistin resistance was absent in all isolates.

Detailed antibiotic resistance patterns of gram-negative pathogens isolated from effluent samples from the SPFs are provided in Supplementary Table S2a,b.

### 3.5. Coliforms Bacterial Load in Potable Water Used for Seafood Processing

**Figure 2:**
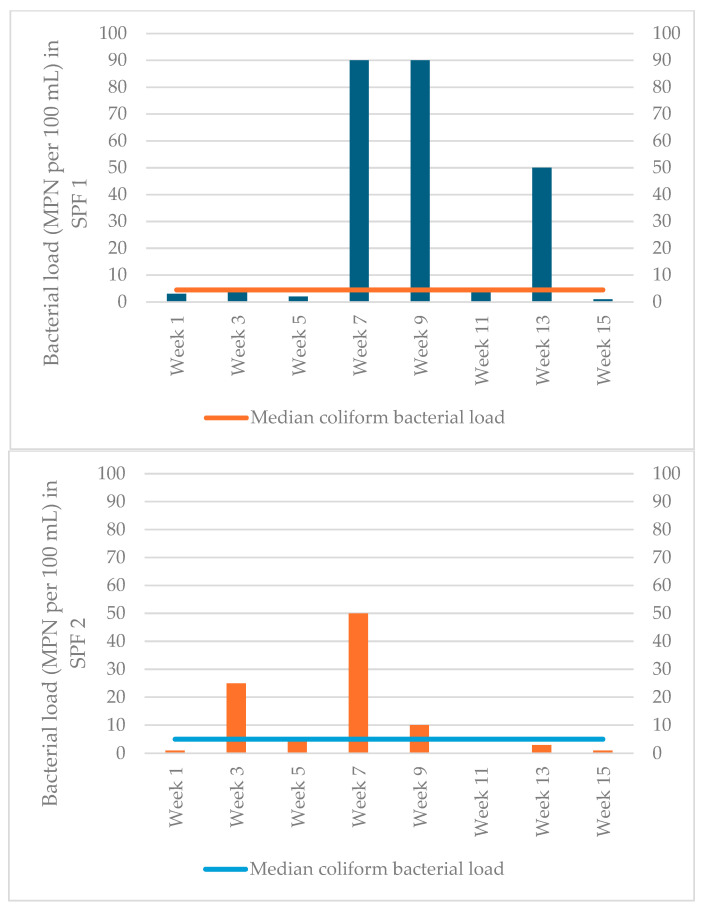
Median coliform bacterial load in the potable water (water used for seafood processing) samples collected from the two seafood processing facilities in Tema, Ghana, during March–November 2024. Abbreviations: MPN: Most probable number; SPF: Seafood processing facilty.

The median coliform bacterial load (IQR) in potable water samples collected from SPF-1 was 4.5 (2.5–70) MPN per 100 mL (Figure 2). However, intermittent elevations were observed, with higher coliform counts recorded in Weeks 7 and 9 (approximately 90 MPN/100 mL) and Week 13 (approximately 50 MPN/100 mL).

The median coliform bacterial load (IQR) in SPF-2 was 5 (1–25) MPN per 100 mL (Figure 2). However, intermittent elevations were observed, with higher coliform counts recorded in Week 3 (approximately 25 MPN/100 mL) and Week 7 (approximately 50 MPN/100 mL), with lower peaks observed in Weeks 9 and 13 (≤10 MPN/100 mL).

The median coliform bacterial load (IQR) in potable water samples collected from SPF-1 was 4.5 (2.5–70) MPN per 100 mL. The median coliform bacterial load (IQR) in SPF-2 was 5 (1–25) MPN per 100 mL (Figure 2).

## 4. Discussion

This study showed a significant reduction in coliform bacterial load in effluents released from the two SPFs in Tema, Ghana, following interventions to improve effluent treatment systems. The coliform bacterial load in both SPFs was reduced by 96% between 2021–2022 and 2024. Despite these reductions in coliform bacterial load, gram-negative and MDR bacteria continue to be detected in the effluents from both SPFs in varying proportions. The potable water samples from both SPFs showed a median coliform bacterial load of 5 MPN per 100 mL.

These findings are particularly relevant in the context of environmental and public health monitoring, as they provide supporting evidence that strategic OR is associated with industry-level changes.

This study offers critical evidence for evaluating progress in effluent management and highlights persistent AMR-related risks in the booming Ghanaian fisheries sector. Moreover, this study is timely and policy-relevant, as Ghana is currently in the process of reviewing and updating its National Action Plan on AMR. Importantly, to the best of our knowledge, there is limited published data from the African region specifically addressing AMR in the seafood processing industry. This research, therefore, helps fill a critical knowledge gap and may serve as a reference for similar interventions across the region and beyond.

This study has a few strengths. First, analysis of samples in both time periods of comparison followed standard quality-assured laboratory procedures, ensuring reliability and reproducibility. Second, the WHO priority pathogens were tested, and multiple drug resistance (MDR) was also determined, thus providing an objective assessment of the magnitude of AMR. Third, the follow-up study assessed the coliform bacterial load in potable water used in seafood processing, thus providing a better understanding of the possible sources of AMR in the SPFs. Fourth, the conduct and reporting of this study were as per the Strengthening the Reporting of Observational Studies in Epidemiology (STROBE) guidelines [29].

This study also has a few limitations. First, there is the absence of molecular testing limits understanding into the genetic basis of observed resistance. Second, there are the variations in operational conditions and sampling time between the baseline and follow-up periods. While follow-up sampling took place throughout several seasons, from March to November 2024, baseline sampling took place in May–July 2021 and March–May 2022. Bacterial survival and detection may be impacted by seasonal variations in effluent volume, temperature, and dilution, which could potentially compromise cross-period comparability. Third, modifications to the effluent system caused SPF-1 to operate at a lower output capacity in 2024, which may have led to lower bacterial counts and organic loading regardless of treatment effectiveness. Fourth, isolate-level antimicrobial resistance results are descriptive and may not accurately reflect within-sample heterogeneity, despite the use of a single isolate per species per sample to reduce redundancy. Therefore, rather than being independent prevalence estimates at the sample level, MDR proportions should be understood as representing resistance among recovered isolates. Fifth, the number of effluent samples analyzed in the two time periods is small due to the financial constraints associated with sampling and microbiological testing. Therefore, the changes observed in bacterial loads and resistance patterns are interpreted with caution.

This study has some key findings and implications. Effluent samples collected from the two SPFs showed a significant reduction (by 96%) in coliform bacterial load across both time periods (May 2021–May 2022 and March–November 2024). The ongoing overhaul in effluent treatment systems in SPF-1 and the introduction of new chemical coagulants in SPF-2 as per recommendations of the baseline OR study may have contributed to the decline in coliform bacterial load. On the other hand, reduced production capacity in SPF-1 coupled with potential seasonal variation could also contribute to the 96% reduction in coliform load. Similar reductions (99%) in bacterial load of effluents were reported from a sewage treatment plant in Ghana when measures to improve plant efficiency were put in place [30]. It is encouraging to note that both the SPFs implemented changes in their effluent treatment system in a short span of time after the baseline OR study. These functional efficacies of these systems should be maintained, to sustain the reduction in coliform bacterial load. These interventions can also serve as models for other food-processing industries.

Gram-negative pathogenic bacteria were present in samples from both SPFs in the follow-up study, though in different proportions [13,19]. The persistence of certain bacteria despite the introduction of improved effluent treatment systems warrants further investigation. In SPF-1, *E. coli* was found in almost all the samples, *Proteus* spp. was found in half of the samples, and *Salmonella* spp. was found in three samples. These are indicators of fecal contamination and may point to the use of contaminated potable water or poor hygiene practices at SPF-1. It is also possible that these hardy resilient organisms possess mechanisms like biofilm-formation that enable survival in disinfected environments [31]. The absence of *Proteus* spp. in SPF-2 across both periods also suggests that site-specific factors, such as effluent handling practices or localized environmental conditions, may influence species distribution. The emergence of *Salmonella* spp. in 15% of samples in SPF-1 and *P. aeruginosa* in 30% of samples in SPF-2 in 2024 indicates a shifting microbial profile and potential new risk which underscores the need for continuous AMR surveillance.

An alarming finding of the baseline OR study was the presence of *Enterobacteriaceae* resistant to third-generation cephalosporins and multidrug-resistant pathogens [19]. It was reassuring to note that these pathogens were isolated from significantly fewer samples compared to the baseline OR study in SPF-1. SPF-2, however, had the same proportion of *Enterobacteriaceae* resistant to third-generation cephalosporins in two time periods. This could be due to a number of factors such as persistence of resistant strains in the environment, resulting in biofilms in pipelines and treatment systems harboring resistant bacteria and allowing them to persist despite intervention [32].

Multidrug resistance (MDR) among gram-negative pathogens generally declined between the two study periods. In SPF-1, the proportion of MDR *E. coli* declined from 63% in 2021–2022 to 32% in 2024, while MDR *K. pneumoniae* decreased slightly from 40% to 25%. In SPF-2, MDR *E. coli* dropped from 67% to 33%, and MDR *K. pneumoniae* significantly decreased from 100% to 56%. However, MDR *P. aeruginosa* was not detected in 2021–2022 but emerged in 2024 with all six isolates exhibiting resistance (100%). Overall, while MDR levels in *E. coli* and *K. pneumoniae* declined, the emergence of highly resistant *P. aeruginosa* highlights a persistent risk in effluent from seafood processing facilities [33].

Overall, resistance levels among gram-negative pathogens isolated from effluent in both seafood processing facilities declined between 2021–2022 and 2024, particularly in *E. coli* and *K. pneumoniae*. Reductions in resistance to tetracyclines, trimethoprim–sulfamethoxazole, and third-generation cephalosporins suggest potential improvements in antimicrobial stewardship or changes in effluent management practices that may have reduced selective pressure in the aquatic environment. Nevertheless, the emergence of carbapenem resistance in *K. pneumoniae* and *A. baumannii* in 2024 is concerning, as it may reflect the introduction of resistant strains from clinical or community sources or selective survival under environmental stressors. The absence of colistin resistance is encouraging, but the detection of carbapenem non-susceptibility underscores the risk of last-line agents being compromised and highlights the importance of sustained surveillance and interventions to mitigate the spread of highly resistant bacteria through industrial effluents [34].

This study was the first to assess the coliform bacterial load in potable water used in the two SPFs. The median bacterial load in both SPFs was approximately 5 MPN per 100 mL. Ideally, potable water should be devoid of any bacteria. There was a variation in the coliform bacterial load across samples collected on different days within the same facility. Since both facilities source water from Ghana Water Limited, this variation likely stems from post-supply contamination within facility infrastructure, e.g., unclean tanks, ageing pipes, cross-contamination with non-potable water pipes, or poor storage practices. According to WHO drinking water guidelines and water resource commission drinking water guidelines in Ghana, potable water used for human consumption and food processing should be free of detectable coliform bacteria (0 MPN/100 mL). Therefore, the detection of coliforms in potable water used within the seafood processing facilities, although at low levels, indicates non-compliance with recommended standards and suggests a potential source of microbial contamination within the production environment.

Based on the findings, the following recommendations can be put forward. To tackle hardy bacteria, although not evaluated in the present study, previous studies have reported that multi-step coagulation–flocculation approaches, such as ferric chloride followed by polymer-assisted flocculation, may enhance the removal of microbial contaminants from industrial effluents. Such strategies warrant further investigation in seafood processing facilities to determine their effectiveness in reducing bacterial loads and antimicrobial resistance.

Ferric chloride is more effective as a primary coagulant due to its strong destabilization and removal of suspended solids and organics. Polyacrylamides add flocculation efficiency and significantly reduce sludge volumes when used after ferric chloride [35]. Considering the detection of bacterial load in potable water, measures to disinfect potable water prior to use, like chlorination, should be implemented in the SPFs. There is a need to assess and upgrade onsite water storage and distribution systems to prevent post-supply contamination. Coliform bacterial load measurement and priority pathogen surveillance in potable water samples should be carried out in coordination with the Council for Scientific and Industrial Research-Water Research Institutes. The persistent or emerging resistance in *P. aeruginosa*, *A. baumannii*, and certain *K. pneumoniae* isolates underscores the need for continued surveillance to identify and address the emergence of new patterns of resistance. Furthermore, AMR surveillance should be systematically extended to inland aquaculture facilities that utilize freshwater sources for seafood production.

## 5. Conclusions

Although lower bacterial loads were observed following operational changes, other contributing factors cannot be excluded; the persistence of specific pathogenic bacteria underscores the need for full implementation of the recommendations of the baseline study. In particular, the establishment of in-house potable water treatment and disinfection systems, coupled with efficient and consistently monitored effluent treatment processes, is critical to prevent re-contamination within production systems. Furthermore, the detection of WHO-priority pathogens highlights the necessity for integrated antimicrobial resistance (AMR) surveillance that links potable water, production environments, and effluent discharges. Such an integrated approach is essential to sustain current gains, mitigate the dissemination of resistant pathogens into receiving aquatic ecosystems, and protect public health. Future research employing season-matched sampling, production-normalized bacterial burdens, and concurrent control facilities would enhance the robustness of causal inferences.

## Figures and Tables

**Table 1:** Number and proportion of effluent samples collected from the two seafood processing facilities (SPF) in Tema, Ghana which contained gram-negative pathogens during May 2021–May 2022 compared to March–November 2024.

Bacterial Species	Seafood Processing Facility 1					Seafood Processing Facility 2				
	May 2021–May 2022 ^1^ (N = 19)		Mar–Nov 2024 (N = 20)		*p*-Value	May 2021–May 2022 ^1^ (N = 19)		Mar–Nov 2024 (N = 20)		*p*-Value
	*n*	(%) ^2^	*n*	(%) ^2^		*n*	(%) ^2^	*n*	(%) ^2^	
*Escherichia coli*	16	(84)	19	(95)	0.565 ^3^	15	(79)	3	(15)	**<0.001** ^3^
*Klebsiella pneumoniae*	5	(26)	4	(20)	0.716 ^3^	10	(53)	9	(45)	0.617 ^4^
*Proteus* spp.	6	(32)	10	(50)	0.253 ^4^	0	(0)	0	(0)	-
*Acinetobacter baumannii*	0	(0)	0	(0)	-	2	(11)	1	(5)	0.605 ^3^
*Pseudomonas aeruginosa*	2	(11)	0	(0)	0.231 ^3^	0	(0)	6	(30)	**0.020 ^3^**
*Salmonella* spp.	0	(0)	3	(15)	0.231 ^3^	0	(0)	0	(0)	-

^1^ Data for the period May 2021–May 2022 has been published and can be accessed at https://doi.org/10.3390/ijerph191710823 (accessed on 22 July 2025) [19]. ^2^ Percentage calculated out of the total number of samples in each time period for each facility. ^3^ *p*-value derived using Fisher’s exact test; values in bold denote statistical significance. ^4^ *p*-value derived using z-test for proportions.

**Table 2:** Number and proportion of effluent samples collected from the two seafood processing facilities (SPF) in Tema, Ghana, which contained multidrug-resistant gram-negative pathogens during May 2021–May 2022, compared to March–November 2024.

Bacterial Species	May 2021–May 2022 ^1^			Mar–Nov 2024			*p*-Value
	Isolates	MDR ^2^		Isolates	MDR ^2^		
		*n*	(%) ^3^		*n*	(%) ^3^	
Seafood processing facility 1							
*Escherichia coli*	16	10	(63)	19	6	(32)	0.067 ^4^
*Klebsiella pneumoniae*	5	2	(40)	4	1	(25)	0.999 ^5^
*Proteus* spp.	6	1	(17)	10	2	(20)	0.999 ^5^
*Acinetobacter baumannii*	0	-	-	0	-	-	-
*Pseudomonas aeruginosa*	2	0	(0)	0	-	-	-
*Salmonella* spp.	0	-	-	3	0	(0)	-
Seafood processing facility 2							
*Escherichia coli*	15	10	(67)	3	1	(33)	0.528 ^5^
*Klebsiella pneumoniae*	10	10	(100)	9	5	(56)	**0.033 ^5^**
*Proteus* spp.	0	-	-	0	-	-	-
*Acinetobacter baumannii*	2	0	(0)	1	0	(0)	-
*Pseudomonas aeruginosa*	0	-	-	6	6	(100)	-
*Salmonella* spp.	0	-	-	0	-	-	-

^1^ Data for the period May 2021-May 2022 has been published and can be accessed at https://doi.org/10.3390/ijerph191710823 (accessed on 22 July 2025) [19]; ^2^ Isolates showing phenotypic resistance to three or more different antimicrobial classes were considered MDR. ^3^ Percentage calculated out of total number of isolates in each time period for each facility. ^4^ *p*-value derived using z-test for proportions. ^5^ *p*-value derived using Fisher’s exact test; values in bold denote statistical significance. Abbreviations: MDR: Multidrug-resistant.

## Data Availability

Requests to access these data should be sent to the corresponding author.

## Supplementary Materials

The following supporting information can be downloaded at https://www.mdpi.com/article/10.3390/life16010107/s1, Figure S1: Representative figure for Kirby-Bauer Disk Diffusion Assay Agar Plate; Figure S2: Representative figure for ESBL; Table S1: Dissemination Details of the baseline Operational Research Study; Table S2a,b: Antibiotic resistance pattern of gram-negative pathogens isolated from effluent samples collected from the two SPFs understudy between the two time periods; File S1: Supplementary file on the MALDI-TOF Spectra; Laboratory Test Procedure for coliform enumeration.

## Abbreviations

The following abbreviations are used in this manuscript:

AMR	Antimicrobial Resistance
CSIR	Council for Scientific and Industrial Research
CLSI	Clinical Laboratory Standards Institute
DAF	Dissolved Air Flotation
EMB	Eosin Methylene Blue
EPA	Environmental Protection Authority
ESBL	Extended Spectrum β-Lactamase
GAP	Global Action Plan
GWL	Ghana Water Limited
IQR	Interquartile Range
LMIC	Low- and Middle-Income Country
MALDI-TOF	Matrix-Assisted Laser Desorption/Ionization Time-of-Flight
MAR	Multiple Antibiotics Resistance
MDR	Multidrug Resistance
MPN	Most Probable Number
OR	Operational Research
PAM	Polyacrylamide
SORT IT	Structured Operational Research and Training Initiative
SPF	Seafood Processing Facilities
SPF-1	Seafood Processing Facility-1
SPF-2	Seafood Processing Facility-2
STP	Sewage Treatment Plant
STROBE	Strengthening the Reporting of Observational Studies in Epidemiology
WHO	World Health Organization
EDTA	Ethylenediaminetetraacetic Acid
CIM	Carbapenem Inactivation Method
eCIM	EDTA-Modified Carbapenem Inactivation Method
MBL	Metallo-β-Lactamase
CDT	Combination Disk Test
