# Supplementary material for: Coliform Load and Antimicrobial Resistance in Ghana’s Seafood Processing Effluent (2021–2024): Evidence of Operational Improvement and Persistent AMR Risk
Source: Life (Basel). 2026 Jan 12;16(1):107. doi: 10.3390/life16010107 (PMC12843266; doi:10.3390/life16010107)
Supplement: Supplementary file 1 [file life-16-00107-s001.zip › Laboratory Test Procedure for Coliform Enumeration.pdf]

Laboratory Test Procedure

For each effluent sample, three rows of five sterile universal bottles were arranged in a test-tube rack, giving a total of 15 bottles per sample. Using a sterile pipette, 10 mL of double-strength Lauryl Tryptose Broth (LTB) was dispensed into each bottle in the first row, while 10 mL of single-strength LTB was dispensed into each bottle in the second and third rows. Subsequently, 10 mL of the undiluted effluent sample was inoculated into each of the five bottles containing double-strength LTB (first row). Using a fresh sterile pipette, 1 mL of the undiluted effluent was inoculated into each of the five bottles containing single-strength LTB (second row). To prepare a 1:10 dilution, 1 mL of effluent was added to 9 mL of sterile distilled water. From this dilution, 1 mL was inoculated into each of the five bottles containing single-strength LTB in the third row.

Inoculated tubes were incubated at 35 ± 0.5 °C for 24 hours, after which they were examined for turbidity, gas production, and acid formation. If no gas or acid production was observed, incubation was extended up to 48 hours. Coliform bacterial load was then estimated using the Most Probable Number (MPN) index and reported as MPN/100 mL.

Positive tubes were sub-cultured by streaking onto MacConkey agar (OXOID, UK) for coliform isolation, and Eosin Methylene Blue (EMB) agar (OXOID, UK) for the isolation of other Gram-negative bacteria. The plates were then incubated at 37 °C for 15–24 hours.

For each positive sample, a single presumptive colony per target gram-negative bacterial species was selected based on colony morphology on selective media and sub-cultured for confirmation and antimicrobial susceptibility testing. Multiple colonies of the same species from a single sample were not included in order to reduce redundancy and minimize non-independence among isolates. Antimicrobial resistance analyses were therefore conducted at the isolate level, with each isolate corresponding to a distinct sample–species combination.

Identification

Resulting bacterial isolates were identified using the BD Bruker MALDI Biotyper CA system (MBT compass for Research and MBT compass IVD for clinical diagnosis -Franklin Lakes,NJ,USA/Bruker Daltonics, Bremen Germany) Matrix-Assisted Laser Desorption Ionization Time-of-Flight (MALDI-TOF) mass spectrometry.
