# Supplementary material for: Coliform Load and Antimicrobial Resistance in Ghana’s Seafood Processing Effluent (2021–2024): Evidence of Operational Improvement and Persistent AMR Risk
Source: Life (Basel). 2026 Jan 12;16(1):107. doi: 10.3390/life16010107 (PMC12843266; doi:10.3390/life16010107)
Supplement: Supplementary file 1 [file life-16-00107-s001.zip › Supplementary file S1 .pdf]

Linear MALDI mass resolution for proteins. >= 800 for m/z=12361 Da (Cytochrome C)

Result : 12361.0 : 814;
Limit : 12361.0 : 800;

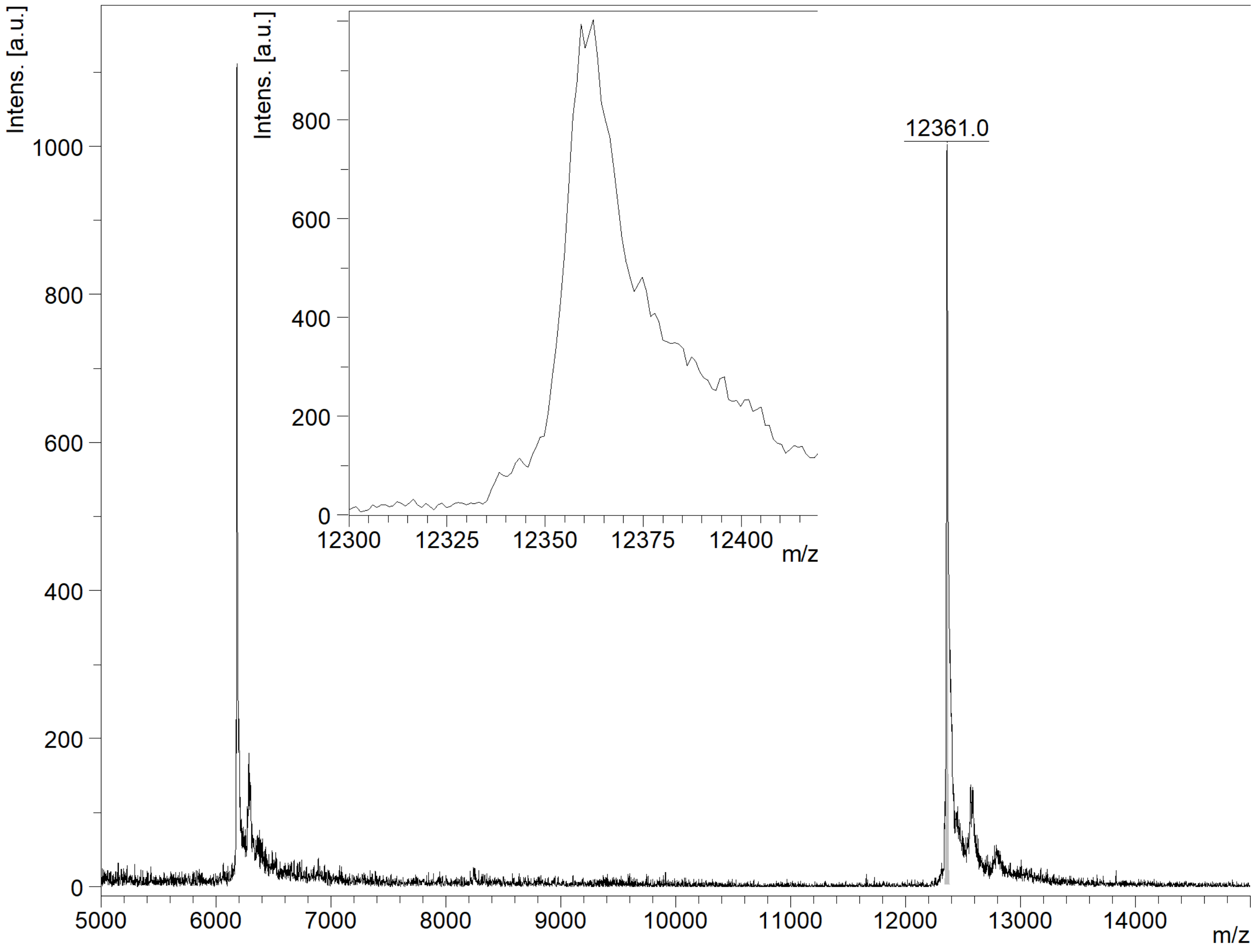

m/z	Res.	S/N
12361.0	814	97

Target

Position	D10

Laser

Laser beam attenuation	75.926
Laser repetition rate	60 Hz
Number of shots	200

Spectrometer

positive voltage polarity	POS
PIE delay	150 ns
Ion source voltage 1	20 kV
Ion source voltage 2	18.2 kV
Lens voltage	6 kV
Linear detector voltage	2.887 kV
Deflection on	
Deflection mass	
SampleRate	2 ns
Reflector voltage 1	0 kV
Reflector detector voltage	0 kV
MSMS parent mass	

Instrument

Instrument type	microflex
Serial instrument number	8604674.04850
Name of computer	MBT-WIN10-LTSC
Operator ID or name	tof-user
flexControl version	flexControl 3.4.206.67
flexAnalysis version	

Date of Acquisition	2025-07-31T15:21:59.215+02:00
Acquisition method	C:\BDalSystemData\Bruker_Service\In_Factory_191022\Methods\flexControlMethods\LP_12kDa.par
Processing method	C:\BDALSystemData\Tools\Spec_check\Methods\Cent_Protein_low.FAMSMethod
File Name	D:\Data\Specs\PC_replacement\LP_12kDa\0_D10\1

printed:7/31/2025 3:22:09 PM
