# Supplementary material for: Coliform Load and Antimicrobial Resistance in Ghana’s Seafood Processing Effluent (2021–2024): Evidence of Operational Improvement and Persistent AMR Risk
Source: Life (Basel). 2026 Jan 12;16(1):107. doi: 10.3390/life16010107 (PMC12843266; doi:10.3390/life16010107)
Supplement: Supplementary file 1 [file life-16-00107-s001.zip › Supplementary table S1.pdf]

Table S1 :Dissemination Details of the Baseline Study

Table S1 shows the type of information about, how, to whom, where, and when, the information on research findings was shared and how many of the dissemination tools were used.

Table S1. Dissemination details of the baseline operational research study conducted by Agyarkwa et al., 2022, Ghana

How	To Whom (Numbers[1])	Where	When
PowerPoint Presentation	Facility Managers of Seafood Processing Facilities (3)<br>The focal person on AMR at Environmental Protection Authority EPA (1)<br>Select staff of EPA (4)	EPA Head Office, Accra	March 2023
PowerPoint Presentation	Key stakeholders of the One Health platform in Ghana (12)<br>WHO Country Office Representative (1)<br>Fellow Ghana SORT-IT Alumni (11)	Oak Plaza Hotel, Accra	July 2023
Publication upload	Researchers/Journal readers (2330 views and six citations as of 22 July 2025)<br>Followers of social media accounts and general viewers	Journal<br><br>LinkedIn<br>WhatsApp	July 2023
Poster Presentation exhibition on World Environment Day	Hon. Minister of Environment, Science, Technology, and Innovation of the Government of Ghana<br>Staff of EPA<br>Non-governmental organizations<br>General Public<br>The media	EPA Head Office, Accra	June 2024

Dissemination materials included a published article, a plain language handout, a ten-minute technical presentation and any other material; [1]The number of individuals attending the meeting.

Abbreviations: AMR – Antimicrobial Resistance
