# Supplementary material for: Coliform Load and Antimicrobial Resistance in Ghana’s Seafood Processing Effluent (2021–2024): Evidence of Operational Improvement and Persistent AMR Risk
Source: Life (Basel). 2026 Jan 12;16(1):107. doi: 10.3390/life16010107 (PMC12843266; doi:10.3390/life16010107)
Supplement: Supplementary file 1 [file life-16-00107-s001.zip › Supplementary table S2a and b .pdf]

**Supplementary Table S2a.** Antibiotic resistance patterns of gram-negative pathogens isolated from effluent samples collected from a seafood processing facilities (SPF-1) in Tema, Ghana during May 2021-May 2022 compared to March-November 2024

| **Antibiotics** | **Isolates Resistant to Antibiotics** | | | | | | | | | | | |
|---|---|---|---|---|---|---|---|---|---|---|---|---|
| | ***Escherichia coli*** | | | | ***Klebsiella pneumoniae*** | | | | ***Proteus* spp.** | | | |
| | May 2021-May 2022[1] (N=16) | | Mar-Nov 2024 (N=19) | | May 2021-May 2022[1] (N=5) | | Mar-Nov 2024 (N=4) | | May 2021-May 2022[1] (N=6) | | Mar-Nov 2024 (N=10) | |
| | n | (%)[2] | n | (%)[2] | n | (%)[2] | n | (%)[2] | n | (%)[2] | n | (%)[2] |
| **Access group** | | | | | | | | | | | | |
| Tetracycline | 13 | (81) | 5 | (26) | 0 | (0) | 0 | (0) | 3 | (50) | 5 | (50) |
| Sulfamethoxazole /Trimethoprim | 9 | (56) | 4 | (21) | 1 | (20) | 0 | (0) | 0 | | 2 | (20) |
| Gentamicin | 0 | (0) | 2 | (11) | 0 | (0) | 1 | (25) | 0 | (0) | 0 | (0) |
| Ampicillin | 10 | (63) | 8 | (42) | 4 | (80) | 0 | (0) | 3 | (50) | 4 | (40) |
| Amikacin | 2 | (13) | 2 | (11) | 0 | (0) | 0 | (0) | 0 | (0) | 0 | (0) |
| Amoxicillin | - | - | 2 | (11) | - | (-) | 2 | (50) | - | (-) | 1 | (10) |
| **Watch group** | | | | | | | | | | | | |
| Cefuroxime | 12 | (75) | 14 | (74) | 4 | (80) | 1 | (25) | 2 | (33) | 6 | (60) |
| Cefotaxime | 6 | (38) | 1 | (5) | 0 | (0) | 0 | (0) | 0 | (0) | 0 | (0) |
| Ciprofloxacin | 3 | (19) | 3 | (16) | 0 | (0) | 0 | (0) | 0 | (0) | 0 | (0) |
| Levofloxacin | 5 | (31) | 1 | (5) | 0 | (0) | 0 | (0) | 0 | (0) | 0 | (0) |
| Ceftazidime | 0 | (0) | 0 | (0) | 0 | (0) | 0 | (0) | 0 | (0) | 0 | (0) |
| Piperacillin/Tazobactam | 5 | (31) | 2 | (11) | 0 | (0) | 1 | (25) | 0 | (0) | 0 | (0) |
| Cefepime | 3 | (19) | 1 | (5) | 1 | (20) | 0 | (0) | 0 | (0) | 0 | (0) |
| Meropenem | 1 | (6) | 0 | (0) | 0 | (0) | 1 | (25) | 0 | (0) | 0 | (0) |
| Ceftriaxone | 0 | (0) | 0 | (0) | 1 | (20) | 0 | (0) | 0 | (0) | 0 | (0) |
| **Reserve group** | | | | | | | | | 0 | (0) | | |
| Colistin | 0 | (0) | 0 | (0) | 0 | (0) | 0 | (0) | 0 | (0) | 0 | (0) |

**Supplementary Table S2b.** Antibiotic resistance patterns of gram-negative pathogens isolated from effluent samples collected from a seafood processing facilities (SPF-2) in Tema, Ghana during May 2021-May 2022 compared to March-November 2024

| **Antibiotics** | **Isolates Resistant to Antibiotics** | | | | | | | | | | | |
|---|---|---|---|---|---|---|---|---|---|---|---|---|
| | ***Escherichia coli*** | | | | ***Klebsiella pneumoniae*** | | | | ***Acinetobacter baumannii*** | | | |
| | May 2021-May 2022[1] (N=15) | | Mar-Nov 2024 (N=3) | | May 2021-May 2022[1] (N=10) | | Mar-Nov 2024 (N=9) | | May 2021-May 2022[1] (N=2) | | Mar-Nov 2024 (N=1) | |
| | n | (%)[2] | n | (%)[2] | n | (%)[2] | n | (%)[2] | n | (%)[2] | n | (%)[2] |
| **Access group** | | | | | | | | | | | | |
| Tetracycline | 10 | (67) | 0 | (0) | 9 | (60) | 3 | (33) | 0 | (0) | 0 | (0) |
| Sulfamethoxazole/Trimethoprim | 8 | (53) | 0 | (0) | 9 | (60) | 3 | (33) | 0 | (0) | 0 | (0) |
| Gentamicin | 0 | (0) | 0 | (0) | 0 | (0) | 0 | (0) | 0 | (0) | 0 | (0) |
| Ampicillin | 13 | (87) | 2 | (67) | 10 | (67) | 9 | (100) | 2 | (13) | 1 | (100) |
| Amikacin | 0 | (0) | 1 | (33) | 0 | (0) | 3 | (33) | 0 | (0) | 0 | (0) |
| Amoxicillin | - | (-) | 0 | (0) | - | (0) | 4 | (44) | - | (-) | | |
| **Watch group** | | | | | | | | | | | | |
| Cefuroxime | 15 | (100) | 2 | (67) | 10 | (67) | 6 | (67) | 2 | (13) | 1 | (100) |
| Cefotaxime | 2 | (13) | 0 | (0) | 0 | (0) | 2 | (22) | 0 | (0) | 1 | (100) |
| Ciprofloxacin | 2 | (13) | 0 | (0) | 1 | (7) | 1 | (11) | 0 | (0) | 0 | (0) |
| Levofloxacin | 5 | (33) | 0 | (0) | 0 | (0) | 0 | (0) | 0 | (0) | 0 | (0) |
| Ceftazidime | 1 | (7) | 0 | (0) | 0 | (0) | 1 | (11) | 0 | (0) | 1 | (100) |
| Piperacillin/Tazobactam | 5 | (33) | 1 | (33) | 9 | (60) | 3 | (33) | 0 | (0) | 0 | (0) |
| Cefepime | 2 | (13) | 0 | (0) | 0 | (0) | 1 | (11) | 0 | (0) | 0 | (0) |
| Meropenem | 0 | (0) | 0 | (0) | 0 | (0) | 1 | (11) | 0 | (0) | 0 | (0) |
| Ceftriaxone | 0 | (0) | 0 | (0) | 0 | (0) | 1 | (11) | 0 | (0) | 1 | (100) |
| **Reserve group** | | | | | | | | | 2 | (13) | | |
| Colistin | 0 | (0) | 0 | (0) | 0 | (0) | 0 | (0) | | | 0 | (0) |

[1]Data for the period May 2021-May 2022 has been published and can be accessed at https://doi.org/10.3390/ijerph191710823 (accessed on 22 July 2025)

[2]Percentage calculated out of total number of isolates in each time period for each facility

N = Number of effluent samples that contained the corresponding bacterial species.
