# Supplementary figures and images for: Coliform Load and Antimicrobial Resistance in Ghana’s Seafood Processing Effluent (2021–2024): Evidence of Operational Improvement and Persistent AMR Risk
Source: Life (Basel). 2026 Jan 12;16(1):107. doi: 10.3390/life16010107 (PMC12843266; doi:10.3390/life16010107)

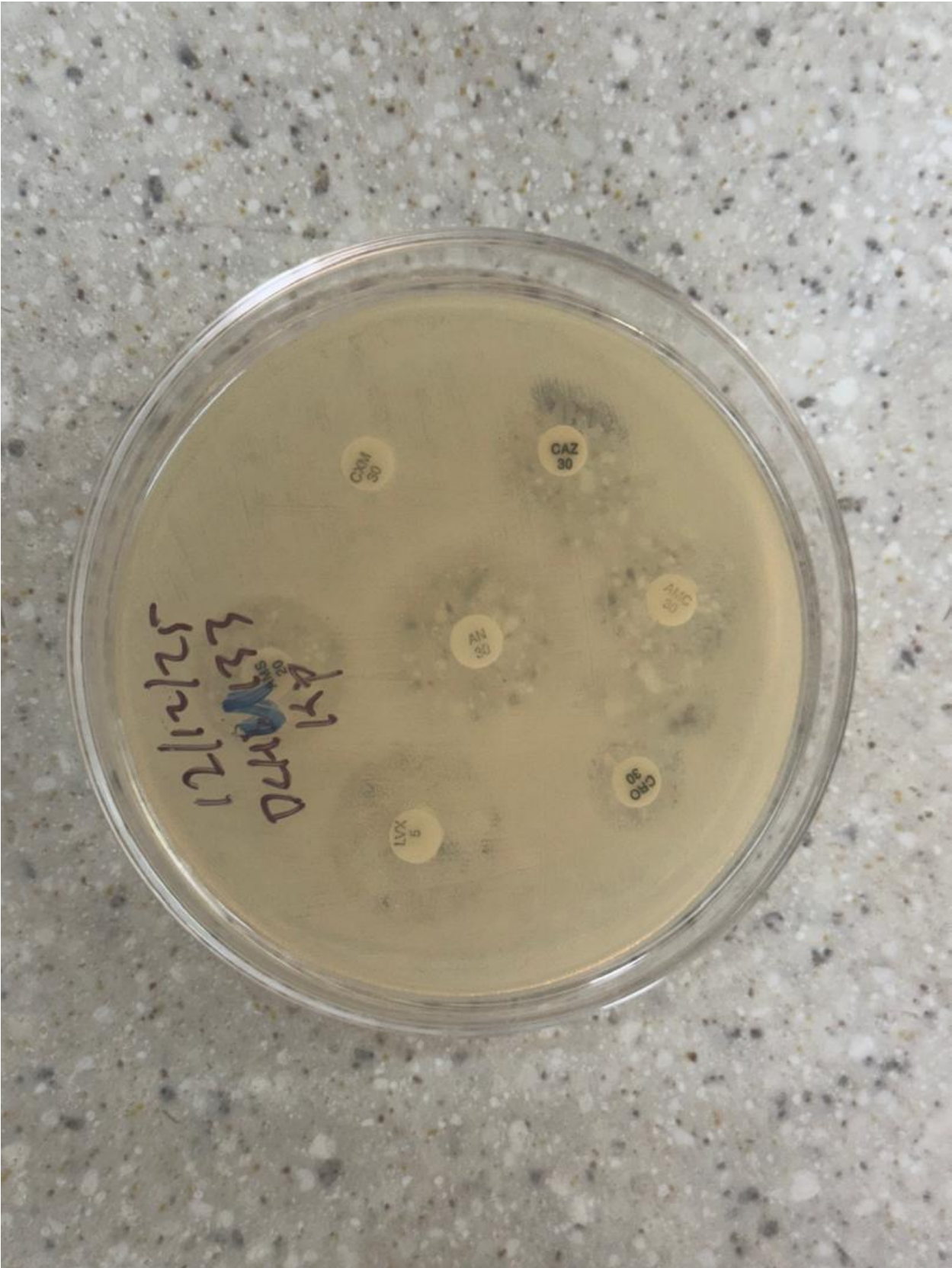

Supplement: Supplementary file 1 [file life-16-00107-s001.zip › Supplementary figure S1.pdf]

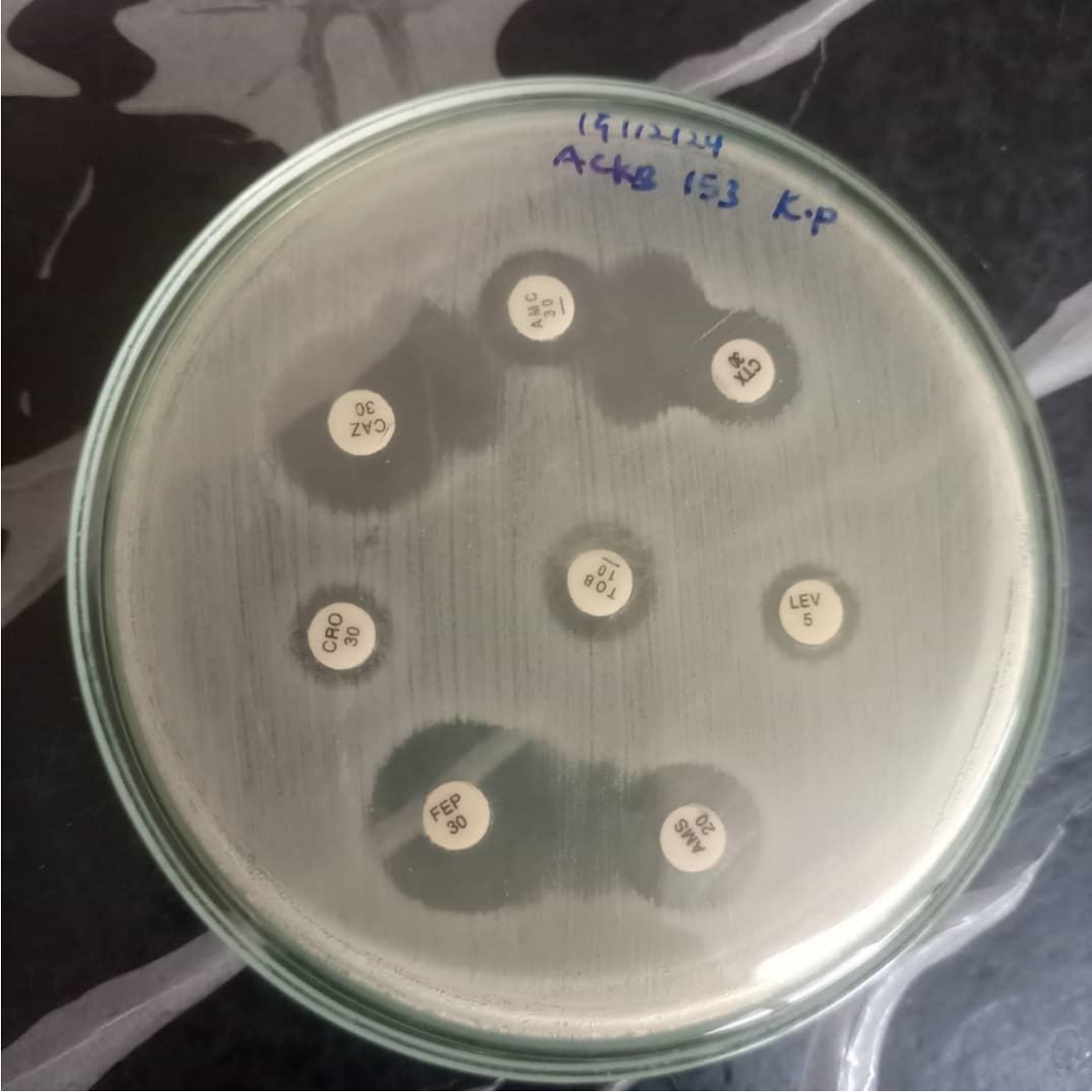

Supplement: Supplementary file 1 [file life-16-00107-s001.zip › Supplementary figure S2 for ESBL.pdf]
